# Nanostructure Formation on Diamond-Like Carbon Films Induced with Few-Cycle Laser Pulses at Low Fluence from a Ti:Sapphire Laser Oscillator

**DOI:** 10.3390/nano8070535

**Published:** 2018-07-16

**Authors:** Seiya Nikaido, Takumi Natori, Ryo Saito, Godai Miyaji

**Affiliations:** Department of Applied Physics, Tokyo University of Agriculture and Technology, 2-24-16 Nakacho, Koganei, Tokyo 184-8588, Japan; nikaidada573@gmail.com (S.N.); impossible.is.nothing.1111@gmail.com (T.N.); s188097x@st.go.tuat.ac.jp (R.S.)

**Keywords:** femtosecond laser, laser ablation, nanostructure formation, surface plasmon polaritons, near-field, diamond-like carbon

## Abstract

This study reports the results of experiments on periodic nanostructure formation on diamond-like carbon (DLC) films induced with 800 nm, 7-femtosecond (fs) laser pulses at low fluence from a Ti:sapphire laser oscillator. It was demonstrated that 7-fs laser pulses with a high power density of 0.8–2 TW/cm^2^ at a low fluence of 5–12 mJ/cm^2^ can form a periodic nanostructure with a period of 60–80 nm on DLC films. The period decreases with increasing fluence of the laser pulses. The experimental results and calculations for a model target show that 7-fs pulses can produce a thinner metal-like layer on the DLC film through a nonlinear optical absorption process compared with that produced with 100-fs pulses, creating a finer nanostructure via plasmonic near-field ablation.

## 1. Introduction

Superimposed femtosecond (fs) laser pulses can form a periodic nanostructure (PNS) on solid surfaces through ablation, where the period size *d* is typically 10–20% of the laser wavelength *λ* [1,2,3,4,5,6]. There has been considerable interest in this surface phenomenon for application in laser nanoprocessing, beyond the diffraction limit of light. Numerous studies have been conducted to understand the mechanism responsible for PNS formation [7,8,9,10]. The experimental conditions and laser parameters for PNS formation have been identified for various target materials, and the dominant physical mechanisms responsible for nanostructuring have been determined.

Based on a series of experiments and model calculations, Miyazaki and Miyaji found that PNS formation is induced by fs laser pulses at a moderate fluence *F* through: a bonding structure change in the material [11,12,13]; generation of high-density electrons on the target surface, leading to the formation of a metal-like layer through linear and nonlinear optical absorption [13,14,15]; near-field ablation around the corrugated nanosurface [13,14,15]; and excitation of standing surface plasmon polariton (SPP) waves [9,10,15,16,17]. These laser–matter interaction processes can explain the origin and growth of PNSs on diamond-like carbon (DLC) [15], Si [16], GaN [10,17], Ti, and stainless steel [9], and theoretical calculations agree well with the observed nanoperiod, which is much smaller than *λ*/2. Based on the physical mechanism, control methods for the PNS shape have been developed, allowing the formation of homogeneous nanogratings [9,10,17] and a saw-like PNS [18] in air. However, some important processes for PNS formation are still unknown, and there is no consensus regarding the detailed mechanism.

For various kinds of material, it has been reported that the *d* value for a PNS increases with increasing *F* for the fs laser pulses at a fluence *F* of a few 100 mJ/cm^2^ to a few J/cm^2^ with a power density *I* of a few TW/cm^2^ [7,8]. Previous studies have concluded that this increase is attributed to the increasing thickness of the metal-like layer produced on the target material with increasing *F* [16,17]. However, this has never been experimentally confirmed. 

The proposed mechanism of PNS formation suggests that a thin metal-like layer can be produced by the fs pulses at low *F* with *I* ~ TW/cm^2^ via a nonlinear absorption process, allowing confirmation of the thickness effect for nanostructuring. In this paper, we report the experimental results of PNS formation on DLC films irradiated with 800 nm, 7-fs laser pulses with a high power density *I* of 0.8–2 TW/cm^2^ and a low fluence *F* of 5–12 mJ/cm^2^ delivered from a laser oscillator. The results indicate the formation of a PNS with a period of *d* = 60–80 nm that *decreases* with increasing *F*. Based on the experimental results and a model calculation, it is shown that the excitation of SPPs at the interface between the thin metal-like layer and the DLC is certainly responsible for the nanostructuring process, and that the decrease of *d* is attributed to the decreasing wavelength of the SPPs with increasing *F* through an increase of electron density in the thin metal-like layer.

## 2. Experimental

**Figure 1:**
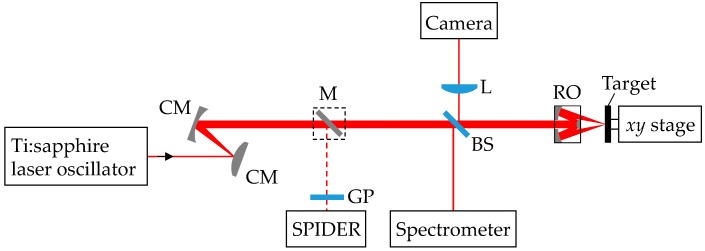
Schematic diagram of optical configuration for nanostructure formation. CM, convex or concave mirror; M, mirror; GP, glass plate; BS, beam splitter; L, lens; RO, reflective objective.

Figure 1 shows a schematic diagram of the optical configuration used in the ablation experiments. As fs laser pulses with a high power density *I* at low fluence *F* can produce a thin metal-like layer on a target surface, the output of a Ti:sapphire laser oscillator was used in the experiments. The pulse duration Δ*τ* was ~7 fs, the wavelength *λ* was 680–940 nm, the repetition rate *f*_rep_ was 80 MHz, and the pulse energy *U*_pulse_ was ~5 nJ. The pulses were so-called few-cycle laser pulses, where the electromagnetic field oscillates for a few cycles [19]. The temporal and spectral profiles of the fs pulses were monitored with a spectral phase interferometry for direct electric-field reconstruction (SPIDER) device and a spectrometer, respectively. When measuring the temporal profile, a silver mirror was inserted to propagate the pulses to the SPIDER device. The output just after the oscillator had a negative group delay dispersion, which was compensated for to minimize the pulse duration by passing the beam through a beam splitter (thickness: 1 mm) and a glass plate (thickness: 1 mm). The laser pulses were spatially expanded with a pair of convex and concave silver mirrors and focused onto the target surface with a ×40 Schwarzschild-type reflective objective (numerical aperture: 0.50) to a spot size *w*_0_ of ~2 μm (1/*e*^2^ radius) on the surface, since the group delay dispersion had to be suppressed to obtain laser pulses with a high power density. A CMOS camera was used to image the focused beam on the target surface. The pulse energy *U*_pulse_ just after the objective was measured with a pyroelectric detector, and the peak fluence *F* = 2 *U*_pulse_/(π *w*_0_^2^) and the peak power density *I* = *F*/Δ*τ* of the fs laser pulses on the target surface were estimated.

As the target, we used a DLC film (thickness: 1.7 μm) that was deposited on a polished silicon substrate with a plasma-based ion implantation system. The root-mean-square value of surface roughness was measured to be less than 1 nm with a scanning probe microscope (SPM). The target was set on an *xy* motorized stage, which could move at a constant speed *v* of 0.1–100 μm/s. The surface morphology was observed using a scanning electron microscope (SEM) and the SPM. A two-dimensional Fourier transform was applied to the SPM images to analyze the distribution of the spatial periodicity in the surface structure along the polarization direction. The bonding structure of the target surface irradiated with the fs pulses was analyzed using micro-Raman spectroscopy with a diode-pumped, single-longitudinal-mode, 532 nm laser beam focused with a ×40 objective.

## 3. Results and Discussion

**Figure 2:**
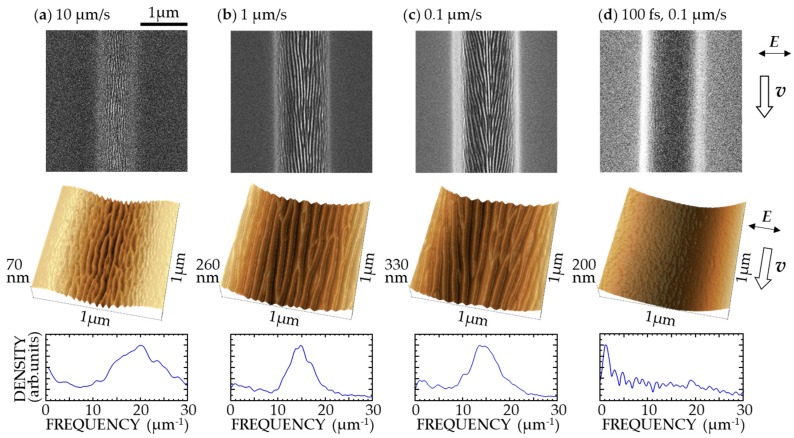
Scanning electron microscopy (SEM) images (**top**), scanning probe microscopy (SPM) images (**middle**), and spatial frequency spectra (**bottom**) of a diamond-like carbon (DLC) film surface irradiated with 7-fs pulses, with *I* = 1 TW/cm^2^ at *F* = 6 mJ/cm^2^ for (**a**) *v* = 10 μm/s, (**b**) *v* = 1 μm/s, and (**c**) *v* = 0.1 μm/s, and (**d**) those irradiated with 100-fs pulses with *I* = 0.1 TW/cm^2^ at *F* = 6 mJ/cm^2^ for *v* = 0.1 μm/s. ***E*** and ***v*** denote directions of polarization and laser scanning, respectively.

Figure 2a–c show SEM and SPM images and spatial frequency spectra of DLC films irradiated with 7 fs pulses with *I* = 1 TW/cm^2^ at *F* = 6 mJ/cm^2^ for *v* = 0.1–10 μm/s. For *v* = 100 μm/s, the surface was observed to swell and was not ablated because of the small shot number of the laser pulses onto the target surface. When *v* was decreased to 10 μm/s (i.e., the shot number increased), the formation of a PNS with a period *d* of ~50 nm was observed on the ablated DLC surface, as shown in Figure 2a. The line-like structure was perpendicular to the direction of polarization. When *v* was decreased to 1 μm/s, a PNS with *d* of ~70 nm formed, as shown in Figure 2b. With a further decrease of *v* to 0.1 μm/s, deeper ablation traces with *d* of ~80 nm formed, as shown in Figure 2c. For comparison, the target surfaces were also irradiated by 100-fs laser pulses with *I* = 0.1 TW/cm^2^ at the same *F*. These pulses were produced by a glass plate (thickness: 3 mm) positioned just after the laser oscillator. As shown in Figure 2d, a PNS did not form on the ablated surface under these conditions.

In previously reported experiments, PNSs formed on DLC films with 100-fs laser pulses with *I* = 1–2 TW/cm^2^ at *F* = 100–200 mJ/cm^2^, delivered from a chirp-pulse amplification Ti:sapphire laser system [3,11,12,13,14,15]. The results shown in Figure 2 suggest possible laser–matter interaction processes for PNS formation, as discussed in previous studies [13,14,15,16]. As *v* is decreased, a bonding structure change—from DLC to glassy carbon (GC)—is induced in the surface layer. This produces nanometer surface roughness due to swelling of the material, as a thin layer with a high electron density is produced on the surface through a nonlinear optical absorption process. On the highly curved swollen metal-like surface, an intense near-field is generated that enhances the incident electric field and initiates nanoscale ablation. Then, SPPs are transiently excited via coherent coupling of the incident laser pulses with the corrugated surface, where the GC layer, including high-density electrons, works as a thin metal layer between air and the DLC for the excitation of SPPs [20]. The periodic enhancement of the near-field of SPPs excited in the surface layer induces ablation, which forms a PNS on the surface. The experimental results shown in Figure 2 indicate that such a process occurs sufficiently when a DLC film is irradiated with 7-fs pulses with a high density of 1 TW/cm^2^ at a low fluence of 6 mJ/cm^2^.

**Figure 3:**
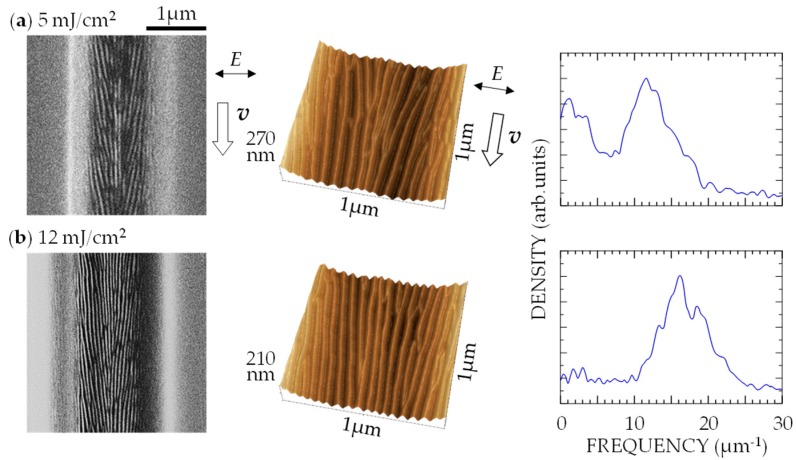
SEM images (**left**), SPM images (**center**), and spatial frequency spectra (**right**) of DLC film surface irradiated with 7-fs pulses at *v* = 0.1 μm/s for (**a**) *I* = 0.8 TW/cm^2^, *F* = 5 mJ/cm^2^ and (**b**) *I* = 2 TW/cm^2^, *F* = 12 mJ/cm^2^. ***E*** and ***v*** denote directions of polarization and laser scanning, respectively.

**Figure 4:**
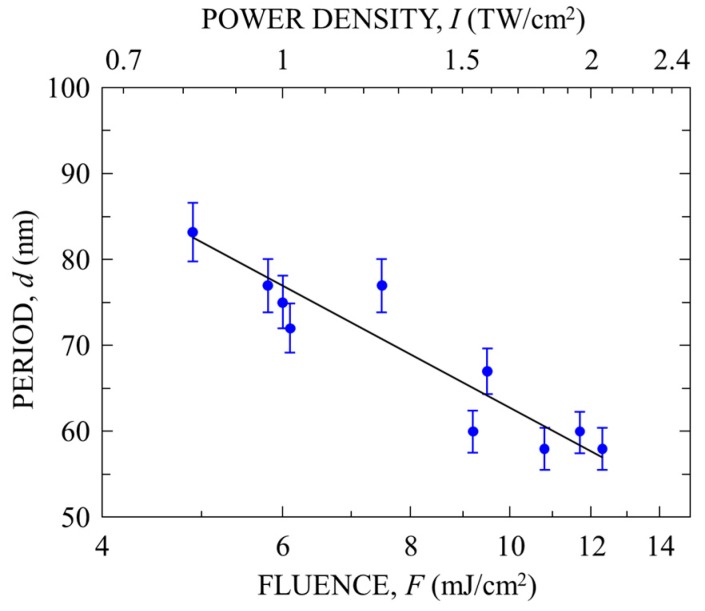
Period *d* of a periodic nanostructure (PNS) on DLC film formed with 7-fs laser pulses as a function of *F* and *I* for *v* = 0.1 μm/s.

An increase in *F* is expected to increase the density of the free electrons produced in the surface layer, leading to a change in surface morphology. To confirm this, surfaces were ablated with 7-fs pulses for *v* = 0.1 μm/s for *F* = 5–12 mJ/cm^2^, corresponding to *I* = 0.8–2 TW/cm^2^. The results are shown in Figure 3. At the lowest *F*, multiple shots produced a PNS with *d* ~85 nm; at the highest *F*, multiple shots produced a finer PNS with *d* ~60 nm. Figure 4 plots the *d* value obtained from the isolated peak position in the Fourier spectrum of the SPM images as a function of *F* and *I*. With increasing *F*, *d* decreases from about 85 to 60 nm. For irradiation with 100-fs laser pulses with *I* = 1–4 TW/cm^2^ at *F* = 100–400 mJ/cm^2^, it has been reported that the *d* value of the PNSs formed on various kinds of material (e.g., DLC, TiN, stainless steel, Ti, Si, and GaN) increased with increasing *F* [3,9,16,17], which is opposite to the results obtained in the present study. This suggests that low-fluence fs pulses with a high power density play a crucial role in the surface morphological change that leads to nanostructuring.

**Figure 5:**
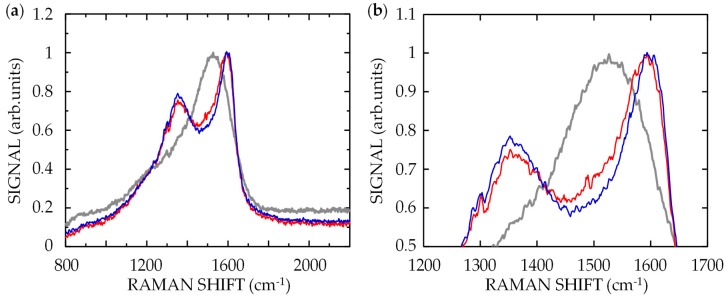
(**a**) Raman spectra of non-irradiated DLC film (gray) and DLC films irradiated with 7-fs (red) and 100-fs (blue) pulses at *F* = 6 mJ/cm^2^ for *v* = 0.1 μm/s; (**b**) expanded spectra of (**a**) in the vicinity of the peaks of D and G bands.

In a previous study, we reported that PNS formation on a DLC surface is preceded by a change in the bonding structure, from DLC to GC [13]. The swelling of the target surface observed for *v* = 100 μm/s indicates that the onset of ablation at *v* ≤ 10 μm/s is preceded by a change in the bonding structure to GC in the target surface. To confirm this, Raman spectra were obtained from surfaces ablated with 7-fs pulses with *I* = 1 TW/cm^2^ at *F* = 6 mJ/cm^2^ for *v* = 0.1 μm/s. The results are shown in Figure 5, together with spectra of surfaces ablated with 100-fs pulses with *I* = 0.1 TW/cm^2^ at *F* = 6 mJ/cm^2^ for *v* = 0.1 μm/s and non-irradiated DLC for comparison. Each spectrum is normalized to give a maximum intensity of unity. The asymmetric broad spectrum for the non-irradiated DLC has a single peak at 1530 cm^−1^, which mainly consists of two spectra at peaks at ~1360 cm^−1^ (D band) and ~1590 cm^−1^ (G band) [21]. The D and G bands are attributed to bond angle disorder in *sp*^2^ graphite-like micro/nanodomains and bond stretching between pairs of *sp*^2^ atoms in both the rings and chains, respectively. The ratio of the intensities of the D and G peaks (*I_D_*/*I_G_*) and the position of the G peak have been reported to indirectly indicate the composition ratio of *sp*^2^ and *sp*^3^ bonding structures in DLC films [22,23,24]. These reports have shown that an increase in *I_D_*/*I_G_* and a shift of the G peak to a higher frequency represent an increase in the amount of *sp*^2^ structures. The spectra from surfaces ablated with 7-fs and 100-fs pulses, shown in Figure 5, clearly show two spectral peaks at 1355 and 1590 cm^−1^, respectively, indicating an increase in disordered carbon or GC [25,26,27,28]. As shown in Figure 5b, *I_D_* for the surface irradiated with 7-fs pulses is smaller than that for the surface irradiated with 100-fs pulses. In addition, the position of the G peak for 7-fs pulses is shifted less than that for 100-fs pulses. These results show that less GC existed in the target surface irradiated with 7-fs laser pulses compared to that which existed with 100-fs pulses, despite the same *F*.

**Figure 6:**
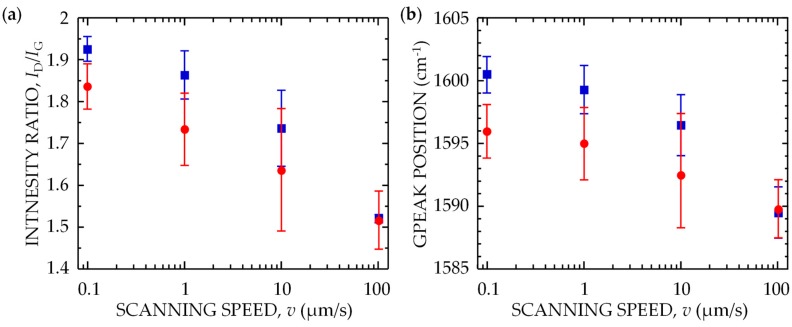
(**a**) Ratio of intensities of D and G peaks (*I_D_*/*I_G_*) and (**b**) position of G peak for DLC films irradiated with 7-fs laser pulses with *I* = 1 TW/cm^2^ at *F* = 6 mJ/cm^2^ (red circles) and those irradiated with 100-fs laser pulses with *I* = 0.1 TW/cm^2^ at *F* = 6 mJ/cm^2^ (blue squares) as function of scanning speed *v*.

To examine the the bonding structural change and ablation processes in detail, Raman spectra were obtained from a DLC film irradiated with 7 fs pulses with *I* = 1 TW/cm^2^ at *F* = 6 mJ/cm^2^ for various values of *v* (*v* = 0.1–100 μm/s). For comparison, spectra were also obtained from a film irradiated with 100 fs pulses with *I* = 0.1 TW/cm^2^ at *F* = 6 mJ/cm^2^. The peak intensities and positions of the D and G bands in the spectra were identified using a curve-fitting program with the Lorentzian function [29]. Figure 6a shows *I_D_*/*I_G_* plotted as a function of *v*. In the spectrum of the non-irradiated DLC film, *I_D_*/*I_G_* was ~1.25. For *v* = 100 μm/s, the ratio for both 7-fs and 100-fs pulses increased to ~1.5. With a decrease in *v*, the ratio monotonically increased, with that for 7-fs pulses being smaller than that for 100-fs pulses. Figure 6b shows the position of the G peak plotted as a function of *v*. In the spectrum of the non-irradiated DLC film, the G peak position was ~1582 cm^−1^. For *v* = 100 μm/s, the position for both 7-fs and 100-fs pulses shifted to ~1590 cm^−1^. With decreasing *v*, the position monotonically shifted to higher frequencies, with that for 7-fs pulses being at lower frequencies than that for 100-fs pulses. These results show two crucial processes for surface modification and subsequent ablation. For *v* = 100 μm/s, where both 7-fs and 100-fs pulses with the same *F* induced only swelling and no ablation on the target, the change in the spectra shown in Figure 6 indicates that the amount of GC at the surfaces irradiated with 7-fs and 100-fs pulses is the same, and that the surface phenomena do not depend on *I*. For *v* ≤ 10 μm/s, where both 7-fs and 100-fs pulses with the same *F* induced not only a bonding structure change but also ablation on the target, the experimental results indicate that 7-fs pulses with higher *I* were strongly absorbed near the target surface through a nonlinear optical absorption process, forming a thinner GC layer than that produced by 100-fs pulses. The surface of the layer was then ablated.

**Figure 7:**
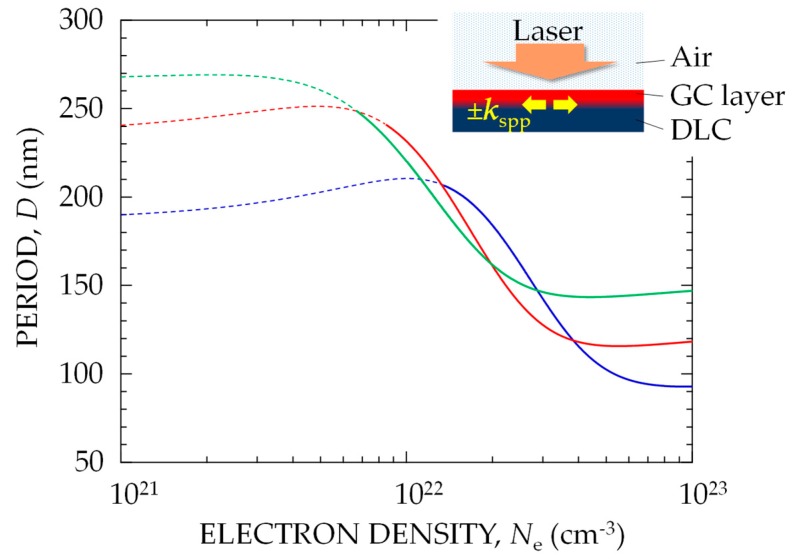
Calculated groove period *D* as function of *N*_e_ in the glassy carbon (GC) layer at *λ* = 600 nm (**blue**), 800 nm (**red**), and 1000 nm (**green**), where the excitation of surface plasmon polaritons (SPPs) is allowed in the region (**solid curves**) of 0 < Re[ε*]. The inset shows a schematic drawing of the initial target surface modeled for calculation. SPPs (**left/right arrows**) are excited at the interface between the DLC and GC layers by a high density of electrons produced by irradiation using high power -density laser pulses (**down arrow**).

Based on these experimental results and the physical mechanism for nanostructuring [8,15,16,30], the origin of the decrease in *d* with increasing *F* is discussed. The SPP wavelength *λ*_spp_ was calculated for the model surface illustrated in the inset of Figure 7, where it was assumed that the fs laser pulses are incident on the target in air, free electrons are produced at the GC surface to form a thin metal-like layer on the DLC substrate, and SPPs are excited at the interface between the metal-like layer and the DLC. The calculation method was almost the same as that used in our previous studies [15,16]. Briefly, *λ*_spp_ = 2π/Re[*k*_spp_] was calculated using the following relation between light and SPPs:

*k*_spp_ = *k*_0_ [*ε*_DLC_ε*/(*ε*_DLC_ + ε*)]^1/2^ (1)

where *k*_0_ is the wavevector of the incident light in vacuum, and ε* and *ε*_DLC_ are the relative dielectric constants for the metallic GC and the DLC, respectively. As the GC layer is ionized by fs laser pulses, ε* rapidly changes during the interaction as:

ε* = *ε*_GC_ − [*ω*_p_^2^/(*ω*^2^ + *iω*/*τ*)] (2)

where *ε*_GC_ is the static dielectric constant for the GC layer, and the second term represents the effect of free electrons with a density of *N*_e_ produced in the GC layer, where *ω* is the laser frequency in vacuum, *τ* = 1 fs is the Drude damping time for free electrons [31,32], and *ω*_p_ = [*e*^2^*N*_e_/(*ε*_0_ m* *m*)]^1/2^ is the plasma frequency, with the dielectric constant of vacuum *ε*_0_, electron charge *e*, electron mass *m*, and optical effective mass of electrons m* = 1. In the calculation, because the wavelength of the 7-fs laser pulse used in the present experiment was 680–940 nm, the static dielectric constants for DLC and GC were used for three wavelengths: *ε*_DLC_ = 6.9 + *i*3.8 and *ε*_GC_ = 3.0 + *i*2.8 for *λ* = 600 nm; *ε*_DLC_ = 8.0 + *i*2.9 and *ε*_GC_ = 3.1 + *i*3.1 for *λ* = 800 nm; and *ε*_DLC_ = 8.5 + *i*2.6 and *ε*_GC_ = 3.6 + *i*4.5 for *λ* = 1000 nm [33].

Figure 7 shows the period of the PNS, *D* = *λ*_spp_/2 = π/(Re[*k*_spp_]), calculated for *λ* = 600, 800, and 1000 nm as a function of *N*_e_. The excitation of SPPs at the interface between the metallic GC layer and the DLC is allowed for Re[ε*] × Re[*ε*_DLC_] < 0 [20], which corresponds to the regions of *N*_e_ > 1.0 × 10^22^ cm^–3^ for *λ* = 600 nm, *N*_e_ > 6.4 × 10^21^ cm^–3^ for *λ* = 800 nm, and *N*_e_ > 5.2 × 10^21^ cm^–3^ for *λ* = 1000 nm. With increasing *N*_e_, *D* decreases from ~200 nm to ~100 nm. Because *N*_e_ should increase with increasing *I* via stronger nonlinear optical absorption, the decrease in *D* with increasing *N*_e_ is in good agreement with the decrease in *d* with increasing *I* for 7-fs laser pulses shown in Figure 4.

The present experimental and calculation results show that the period *d* for a PNS was smaller than *D*, and that the *d* value for a PNS formed with high-fluence 100-fs laser pulses was similar to *D* for *λ* = 800 nm, which is consistent with the results of a previous study [15]. Regarding the excitation of SPPs on a thin metal film, it has been reported that the wavenumber of the SPPs increases with decreasing thickness of the film because of an increase in the radiation damping of SPPs [20]. These results suggest that *d* being smaller than *D* can be attributed to the excitation of SPPs with a larger wavenumber by the thinner metallic layer produced with 7-fs laser pulses. A calculation model for *D* that includes the effect of the metallic layer thickness will be presented and discussed in a separate paper. To discuss the formation process of PNS in detail and make a more accurate model for the nanostructuring, we need to quantitatively measure the amount and thickness of the GC layer on DLC film by using advanced techniques, such as a grazing-incidence small-angle X-ray scattering [34,35,36].

## 4. Conclusions

This study examined the PNS that formed on a DLC film with 7-fs laser pulses at a low fluence from a laser oscillator. The results show the formation of a PNS with a period of *d* = 60–80 nm and a decrease in *d* with increasing fluence. Based on the experimental results and a model calculation, it is shown that the excitation of SPPs at the interface between the thin metal-like layer and the DLC is certainly responsible for the nanostructuring process, and that the decrease of *d* is attributed to the wavelength of the SPPs decreasing with increasing *F* due to an increase of electron density in the thin metal-like layer.
